# Efficacy and Safety of Solitaire Revascularization Device in Managing Refractory Thrombus in Acute Coronary Syndrome

**DOI:** 10.1016/j.jscai.2025.104004

**Published:** 2025-11-11

**Authors:** Deanna Z.L. Khoo, Deborah J.H. Lee, Sanchalika Acharyya, Heng Choon Pooh, Aaron Wong, Poay Huan Loh, Syed Saqib Imran, Yew Seong Goh, Hee Hwa Ho, Fahim H. Jafary, Jason K.K. Loh, Bharat Khialani, Cliff K.F. Li, Randal J.B. Low, Thet Khaing, Eran Sim, Joshua Loh, Paul J.L. Ong

**Affiliations:** a Department of Cardiology, Tan Tock Seng Hospital, Singapore; b Clinical Research & Innovation Office, Tan Tock Seng Hospital, Singapore; c National Heart Centre, Singapore; d National University Heart Centre, Singapore; e Khoo Teck Puat Hospital, Singapore; f Changi General Hospital, Singapore; g Woodlands Health, Singapore; h Capital Heart Centre, Singapore; i Heart Specialist International, Singapore

**Keywords:** acute coronary syndrome, stent retriever, thrombectomy, thrombus

## Abstract

**Background:**

Use of stent retrievers in managing ischemic stroke has been shown to improve outcomes. Data on stent retrievers in managing intracoronary thrombus are limited. This study aimed to assess the safety and efficacy of the Solitaire revascularization device (Medtronic) in managing recalcitrant intracoronary thrombus.

**Methods:**

Patients with acute coronary syndrome and residual large thrombus after treatment with conventional methods underwent treatment with the Solitaire device. The primary efficacy end point was the rate of successful recanalization, defined as Thrombolysis in Myocardial Infarction (TIMI) flow increase to ≥2 or thrombus grade (TG) decrease to ≤2 at postprocedure. Secondary end points included improvements in postprocedure TG and myocardial blush grade. The primary safety end point was occurrence of stroke.

**Results:**

Between June 2019 and November 2022, 51 patients underwent thrombectomy with the Solitaire device. The estimated successful recanalization rate was 76.5% (95% CI, 62.0%-87.2%) after Solitaire use and 92.2% (95% CI, 81.1%-97.8%) at the end of the procedure. TG reduced to ≤2 in 28 (54.9%) patients after Solitaire and 43 (84.3%) patients at the end of the procedure. There was significant improvement in myocardial blush grade with an improvement rate of 40% (95% CI, 22.7%-59.4%) after Solitaire and 66.7% (95% CI, 47.2%-82.7%) at the end of procedure. There was no incidence of stroke postprocedurally or up to 30 days postdischarge.

**Conclusions:**

The Solitaire device is an effective adjunctive tool in managing recalcitrant intracoronary thrombus, with a low risk of complications.

## Introduction

Large intracoronary thrombus in the setting of acute coronary syndrome (ACS) often poses a challenge during percutaneous coronary intervention (PCI), leading to lower procedural success rates. It has been found to increase rates of distal embolization with higher risk of slow or no reflow and subsequent higher risk of mortality and major adverse cardiac events.^1^ Various thrombectomy devices allowing manual or mechanical removal of intracoronary thrombi have been evaluated over past years, with only modest improvements in major cardiovascular events.^2^, ^3^, ^4^ Recent randomized controlled trials have shown no benefit of routine thrombus aspiration on clinical outcomes, and routine aspiration is no longer recommended by current guidelines due to a worrying association with an increased risk of acute ischemic stroke.^5^^,^^6^

Stent retrievers are a novel technology designed to extract organized thrombus from occluded vessels. They were originally created as second-generation mechanical thrombectomy devices with the goal of achieving faster revascularization in patients with acute ischemic stroke.^7^ The most widely used of such devices, the Solitaire revascularization device (Medtronic), has shown superior outcomes vs standard care in terms of recanalization, 90-day mortality, and periprocedural complications in stroke trials.^8^ Although the benefits of using stent retrievers in the neurovasculature is well established, data regarding their use in the coronary vasculature is sparse, with use of the Solitaire device limited to case series and case reports.^9^^,^^10^ A prospective study using the NeVa mechanical thrombectomy device (Vesalio Inc) demonstrated its safety and effectiveness in thrombus removal in patients with ACS as an upfront therapy.^11^ We aimed to examine the efficacy and safety of the Solitaire device as a bailout strategy in the management of refractory thrombus in patients with ACS.

## Materials and methods

### Trial design and patient population

This prospective, multicenter, single-arm feasibility trial enrolled eligible patients from 5 participating tertiary care hospitals in Singapore (ClinicalTrials.gov identifier NCT04692402). Patients enrolled were admitted with ACS with a thrombus burden of grade 4 or above or Thrombolysis in Myocardial Infarction (TIMI) flow grade ≤1 in the infarct-related artery (IRA) recalcitrant to an initial treatment strategy using conventional methods, which included manual, mechanical, or rheolytic thrombectomy, balloon angioplasty, or deferred PCI. Key exclusion criteria included patients ≤21 years, in cardiogenic shock, and those with life expectancy <6 months. Patients with prior stent implanted in the IRA, with significant proximal or ostial stenosis of the IRA or with extensive calcification were also excluded due to the potential difficulty in retrieving the Solitaire device. Patients with bypass grafts as the IRA were also excluded. The study procedure was performed, and patients were followed-up at 30 days postdischarge. The study protocol was approved by all local institutional ethics committees, and written informed consent was obtained from every patient before intervention with the Solitaire device was performed.

### Device description

**Figure 1:**
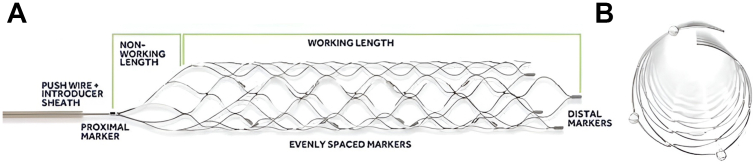
**Solitaire revascularization device.** (**A**) Nitinol stent with proximal and distal platinum markers attached to a push wire. (**B**) Overlapping stent design allows conformation of the stent to vessel walls for thrombus integration into the stent.

The Solitaire revascularization device is a self-expanding nitinol stent with a closed-cell design attached to a push wire (Figure 1). It has a parametric overlapping stent design that allows the device to conform to the vessel wall and optimizes contact with vessel thrombus during deployment. The Solitaire device is available in diameters of 4.0 and 6.0 mm to accommodate vessels of 2.0 to 4.0 mm and 3.0 to 5.5 mm, respectively. Device length range from 20.0 mm to 40.0 mm. Platinum markers at the proximal and distal ends as well as along the stent body allow for easy visualization. In this study, the Solitaire Platinum and Solitaire X generation devices were used. Further device details can be found in Supplemental Table S1.

### Study procedure

Anticoagulation and antiplatelet medications were administered as per institutional practice. Following failure to resolve a large thrombus burden and/or failure to achieve TIMI 3 flow, patients were treated with thrombectomy using the Solitaire device. Selection of the Solitaire device diameter and length was based on the angiographic vessel size and thrombus length. To achieve device delivery, a standard angioplasty wire was used to cross the region of interest. Next, a microcatheter with inner diameter of 0.027 inches was advanced over the angioplasty wire until the end of the microcatheter was positioned distal to the thrombus and the wire was subsequently removed. In its collapsed form, the Solitaire system was delivered through the microcatheter via the introducer sheath using the push wire. Then, the device was advanced until the distal markers line up at the end of the microcatheter. To deploy the device, the push wire was fixed to maintain the position of the device, and the microcatheter was withdrawn proximally allowing the device to expand within the vessel.

**Figure 2:**
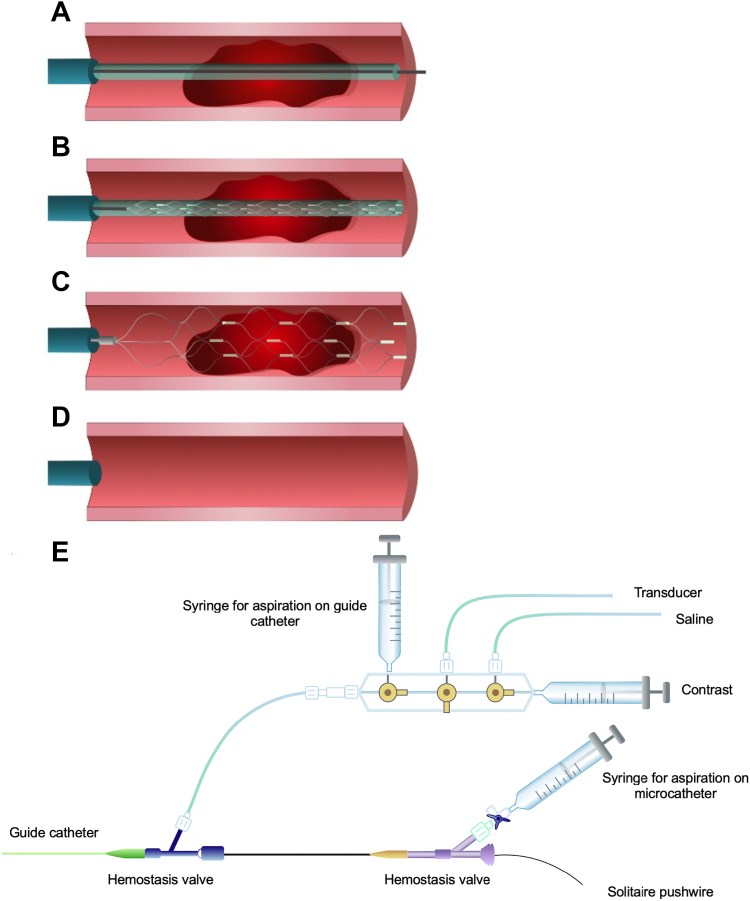
**Deployment of the Solitaire device.** (**A**) Microcatheter is advanced over the wire with the distal tip distal to the thrombus. (**B**) The wire is removed and the Solitaire device loaded into the microcatheter until the distal markers are distal to the thrombus. (**C**) The Solitaire pushwire is held in place while the microcatheter is withdrawn, unsheathing the Solitaire device. (**D**) The Solitaire device and microcatheter are withdrawn together with the integrated thrombus while aspirating on the guide catheter and microcatheter. (**E**) The set up, with syringes on the manifold and microcatheter providing aspiration as the Solitaire device is retrieved.

The device was left in situ for 5 minutes to promote stent thrombus integration. To retrieve the thrombus, the Solitaire device is retracted in its expanded form along with the microcatheter through a guide catheter that is deep seated within the artery. During the retrieval process continuous aspiration was applied both to the guide catheter and to the microcatheter until the device was nearly withdrawn from the guide catheter. The hemostatic valve was then opened for removal of the Solitaire device and microcatheter, and the guide catheter was aspirated to ensure clearance of any thrombus before reconnecting the hemostatic valve back. Figure 2 demonstrates the steps in deployment of the Solitaire device. Operators were limited to 3 recovery attempts per vessel.

### Study end points

#### Efficacy end points

The primary efficacy end point was the rate of successful recanalization, a composite of TIMI flow and thrombus grade (TG). The end point is defined as either TIMI flow increase to ≥2 from a flow of <2 or TG level decrease to ≤2 from >2 at the end of the procedure from any previous assessment point, that is, at the start of procedure, before Solitaire deployment and immediately after Solitaire deployment.

Secondary end points included the successful recanalization rate (as defined earlier) at before and after Solitaire deployment and the individual components of this composite (ie, TIMI score increase and TG decrease after Solitaire deployment and end of the procedure). Additionally, increase in myocardial blush grade (MBG) to ≥2 from a lower value at these assessment points was also evaluated. Refer to Supplemental Table S2 for definitions. Resolution of ST elevation on 12-lead electrocardiogram was assessed at the end of PCI in patients presenting with ST elevation myocardial infarction (STEMI).

Major adverse cardiac and cerebrovascular events was defined as composite of all-cause death, myocardial infarction, target vessel revascularization, or stroke, up to 30 days postdischarge. Procedural complications and cardiovascular events were defined in accordance with the Academic Research Consortium standardized definitions.^12^

#### Safety end points

The primary safety end point was occurrence of death or any stroke defined as the presence of a new focal neurologic deficit thought to be vascular in origin, with signs or symptoms lasting more than 24 hours confirmed by a neurologist and on imaging. Secondary safety outcomes included device-related adverse events, incidence of thrombus embolization in a new territory and all-cause mortality at 30 days postdischarge. A data safety and monitoring board periodically reviewed data related to safety, data integrity, and overall conduct of the trial.

### Statistical analysis

#### Sample size calculation

An a priori sample size calculation was done assuming a desired successful recanalization rate of 35% with Solitaire device and no further interest if the recanalization rate is less than or equal to baseline rate of 18% without Solitaire. At a significance level (α) of .05 and power of 80% (β = 0.20) using Simon optimal design,^13^ we needed to recruit 51 eligible subjects. On the basis of this calculation, 13 subjects with successful recanalization was required at the end of the procedure to determine effectiveness of device.

#### Statistical data analysis

Baseline demographic characteristics and clinical characteristics, procedural details, and clinical outcomes were summarized using appropriate descriptive statistics. Distribution of continuous variables were presented as mean along with SD or median along with IQR. Distribution of categorical variables were presented as frequencies along with percentages. The distribution of TIMI score, MBG, and TG levels across the 4 assessment points were compared using Wilcoxon signed-rank test. Successful recanalization rate, along with MBG and TG improvement rates, were estimated using binomial distribution at immediately after Solitaire deployment and end of the procedure. Clopper–Pearson method was used to compute the 95% CIs. These end points were compared before and after Solitaire deployment using the exact McNemar test. Statistical tests were conducted at 2-sided 5% significance level using SPSS version 26 (IBM Corp).

## Results

### Patient characteristics

**Figure 3:**
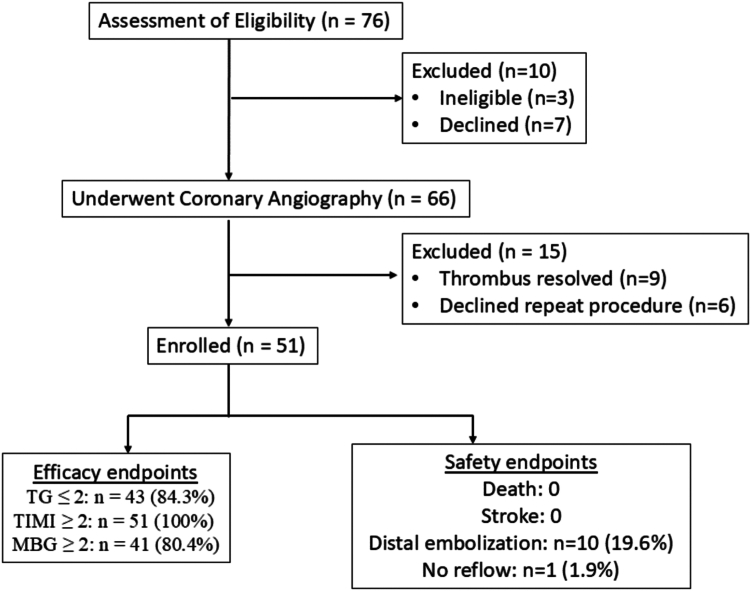
**CONSORT flowchart of study participants’ screening, enrollment and study completion.** MBG, myocardial blush grade; TG, thrombus grade; TIMI, Thrombolysis in Myocardial Infarction.

A total of 51 patients were recruited from 5 participating sites between June 2019 and November 2022. Complete 30-day follow-up data were available for all 51 patients (Figure 3). Baseline demographic characteristics and clinical characteristics of patients are presented in Table 1. Median age of these patients was 57 years, and majority were men (88.2%). More than half of the patients had a history of hypertension (n = 33, 64.7%), and 10 patients (19.6%) had diabetes. Six patients (11.8%) had prior myocardial infarction. Six patients had underwent PCI. The most common symptom at admission was chest discomfort (n = 43, 84.3%). Thirty-eight (74.5%) patients presented with STEMI, 10 (19.6%) patients with non-STEMI, 1 (2%) patient with an evolved myocardial infarction, and 2 (3.9%) patients with unstable angina.

**Table 1:** Distribution of baseline demographic characteristics and clinical characteristics of patients

Characteristic	N = 51
Demographics	
Mean age, y	55.7 ± 10.7
Median age, y	57 (range, 36-81)
Male sex	45 (88.2)
Ethnicity	
Chinese	33 (64.7)
Malay	13 (25.5)
Indian	5 (9.8)
Weight, kg	75.0 (39.1-136.0)
Height, m	1.68 ± 0.08
Body mass index, kg/m^2^	26.8 (16.2-46.0)
Medical history	
Hypertension	33 (64.7)
Type II diabetes mellitus	10 (19.6)
Dyslipidemia	27 (52.9)
Smoking status	
Current smoker (within 1 y)	27 (52.9)
Ex-smoker	6 (11.8)
Family history of premature coronary artery disease	3 (5.9)
History of	
Prior MI	6 (11.8)
Prior percutaneous coronary intervention	6 (11.8)
Prior eart failure	1 (2.0)
Atrial fibrillation	1 (2.0)
Admission features of the patients	
Symptoms	
Chest pain	43 (84.3)
Dyspnea	2 (3.9)
Abdominal pain	1 (2.0)
Pneumonia	1 (2.0)
Missing symptom data	4 (7.8)
Systolic blood pressure, mm Hg	125.04 ± 24.62
Diastolic blood pressure, mm Hg	75.69 ± 15.68
Pulse, bpm	77.00 ± 20.50
Indication for PCI	
ST-elevation MI	38 (74.5)
Non-ST-elevation MI	10 (19.6)
Unstable angina	2 (3.9)
Evolved inferior MI	1 (2.0)

Values are mean ± SD, median (IQR), or n (%) unless specified.

MI, myocardial infarction.

### Procedural characteristics

Table 2 presents the procedural characteristics. The right coronary artery was the culprit vessel in the majority of cases (88.2%). Aspiration was performed for 47 (92.2%) patients. In 8 patients, multiple aspiration devices were used. Refer to Table 2 for the list of devices used. AngioJet was used in 3 patients (5.9%). Glycoprotein IIb/IIIa antagonist was given to 40 patients (78.4%), and majority (41 patients, 80.4%) underwent Solitaire device deployment as deferred strategy (few days after primary procedure involving predilation, thrombus aspiration, and anticoagulation without stent placement).

**Table 2:** Management strategy and procedural details

	N = 51
Intervention details	
Antiplatelet administered prior PCI	48 (94.1)
Procedural anticoagulant	51 (100.0)
Arterial access site	
Radial	47 (92.2)
Femoral	4 (7.8)
Dominance of coronary anatomy	
Right	49 (96.1)
Left	2 (3.9)
Culprit lesion	
Left anterior descending artery	4 (7.8)
Left circumflex artery	2 (3.9)
Right coronary artery	45 (88.2)
Strategy for thrombus management before Solitaire use	
Thrombus aspiration performed	47 (92.2)
Device used for aspiration	
6F Thrombuster II (Kaneka)	23 (48.9)
6F Eliminate (Terumo)	4 (8.5)
6F Export Advance (Medtronic)	4 (8.5)
6F Pronto (Teleflex)	1 (2.1)
7F Pronto (Teleflex)	1 (2.1)
5F Heartrail ST01 (Terumo)	1 (2.1)
CAT RX (Penumbra)	13 (27.6)
Administration of thrombolytic agents	2 (3.9)
Glycoprotein IIb/IIIa antagonist given	40 (78.4)
Deferral of stent implantation	41 (80.4)
Reason for deferred PCI	
Significant thrombus burden	39 (76.5)
Slow flow after balloon predilation	1 (2.0)
No stent needed, ectatic vessels	1 (2.0)
Solitaire device used	
4.0 × 20 mm	1 (2.0)
4.0 × 30 mm	1 (2.0)
4.0 × 40 mm	4 (7.8)
6.0 × 20 mm	2 (3.9)
6.0 × 30 mm	23 (45.1)
6.0 × 40 mm	20 (39.2)
Solitaire vessel size, mm	
≤3.00	3 (5.9)
3.5	10 (19.6)
4	15 (29.4)
>4.0 (4.5-9.0)	22 (43.1)
No. of times device was used	
1	20 (39.2)
2	31 (60.8)

Values are n (%).

PCI, percutaneous coronary intervention.

The Solitaire device was successfully deployed in all cases, with no cases of failure to deploy or retrieve the device. The Solitaire device was deployed once in 19 patients (37.3%) and twice in 31 patients (60.8%). Vessel size ranged from 2.5 to 9.0 mm. The most frequently used Solitaire size was 6.0 × 30.0 mm (n = 23, 45.1%), followed by 6.0 × 40.0 mm (n = 20, 39.2%) (Table 2). Macroscopic thrombus was retrieved from 41 patients (80.4%) (Central Illustration). There were 13 patients (25.5%) with TG 4 to 5 after use of the Solitaire; 8 of these 13 patients had vessel diameters ranging from 5.0 to 9.0mm, and from this group of patients with very large ectatic vessels, 5 patients (mean vessel size 7 mm) had residual thrombus burden of 4 at the conclusion of the procedure. This suggests that the current generation of the Solitaire device is not optimal in large vessel sizes.

**Central Illustration:**
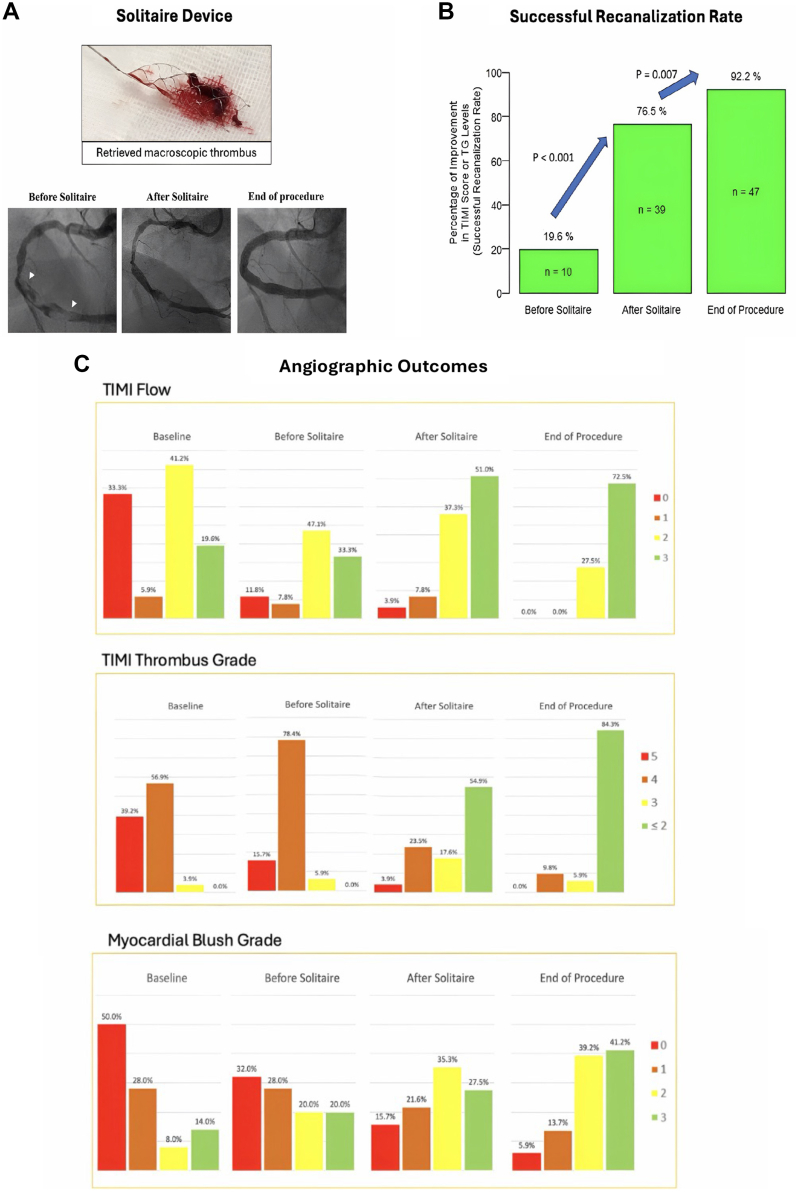
**Solitaire device use and procedural efficacy outcomes.** (**A**) Retrieved macroscopic thrombus. (**B**) A comparison of successful recanalization rate, the composite end point including TIMI score and TG levels, before Solitaire, after Solitaire use, and at the end of the procedure. (**C**) Angiographic outcomes at the start of procedure, before Solitaire, after Solitaire use, and at the end of the procedure. TG, thrombus grade; TIMI, Thrombolysis in Myocardial Infarction.

### End points

#### Efficacy end points

Among the patients included in the study, 20 (39.2%) exhibited TIMI flow grade of <2, 20 (39.2%) displayed TG of >4, and 18 (35.3%) presented with both simultaneously at the start of the procedure in which the Solitaire device was used. The cumulative recanalization rate at the end of the procedure, the primary composite end point, was estimated to be 92.2% (95% CI, 81.1%-97.7%). This was a significant improvement of recanalization rate of 19.6% (95% CI, 9.8%-33.1%; *P* < .001) before Solitaire deployment and 76.5% (95% CI, 62.5%-87.2%; *P* = .007) immediately after Solitaire deployment (Central Illustration). The improvement in recanalization rate immediately after Solitaire was significant too, compared with that before Solitaire (*P* < .001). Recanalization rate did not differ by age or sex groups.

In terms of the individual outcome components, we observed significant improvements in TIMI score and TG levels after Solitaire. Before Solitaire deployment, 10 (19.6%) patients had TIMI flow grade of <2, and 8 (15.7%) displayed TG of >4. Among these 10 patients with TIMI flow grade of <2, 8 achieved grade 2 TIMI flow after Solitaire and all 10 patients achieved either grade 2 or 3 flow by end of the procedure. Of the other 21 patients with grade 2 TIMI flow before the Solitaire deployment, 10 achieved grade 3 TIMI flow after Solitaire and 3 more patients achieved grade 3 TIMI flow by end of the procedure. The distribution of TIMI flow grade over assessment times is displayed in Central Illustration and was found to be significantly different from before to after Solitaire (*P* = .015) and end of procedure (*P* < .001).

Forty-eight patients had TG of ≥4 (40 with grade 4 and 8 with grade 5), before Solitaire deployment; 3 patients had TG of 3. A grade of ≤2 was achieved in 28 (54.9%) patients immediately after using Solitaire and in 43 (84.3%) patients by the end of the procedure. TG improvement rate, defined as lowering thrombus burden to grade of ≤2, was thus estimated to be 54.9% (95% CI, 40.3%-68.9%) immediately after Solitaire and 84.3% (95% CI, 71.4%-93.0%) by the end of the procedure. The distribution of TG values over time is displayed in Central Illustration and was found to be significantly different from before to after Solitaire and end of procedure (*P* < .001). The vessel size in 4 of the 5 patients with TG of ≥4 postprocedurally was large and ranged between 7.0 and 9.0 mm.

Myocardial blush grades 0 and 1 were present in 25 (49%) and 14 (27.5%) patients, respectively, before the procedure. Among these patients, 30 (76.9%) still had MBG of <2 before Solitaire use. This decreased to 18 patients immediately after the Solitaire and to 9 patients by the end of the procedure. MBG improvement rate was thus estimated to be 40% (95% CI, 22.7%-59.4%) immediately after Solitaire and 66.7% (95% CI, 47.2%-82.7%) by the end of the procedure. The distribution of MBG values over time is displayed in Central Illustration and was found to be significantly different from before to after Solitaire and end of procedure (*P* < .001). Among the 38 patients presenting with STEMI, 28 patients (73.7%) demonstrated a resolution of ST elevation of at least 50% from baseline pre-PCI electrocardiogram.

#### Safety end points

There was no occurrence of stroke 24 hours postprocedurally, during admission and up to 30 days postdischarge. There were no cardiac-related and noncardiac-related deaths at up to 30 days postdischarge (Table 3).

**Table 3:** Procedural complications and 30-day outcomes

Outcomes	N = 51
Complications related to PCI	12 (23.5)
Distal embolization	10 (19.6)
No reflow phenomenon	1 (2.0)
Embolization of thrombus from RPL to RPDA	1 (2.0)
Vessel perforation	0
Vessel dissection	0
Inability to deliver or retract Solitaire device	0
Clinical outcomes at 30 days	
Stroke	0
Cardiovascular-related or noncardiovascular-related death	0
Cardiac-related rehospitalization	7 (13.7)
Planned revascularization of nonculprit vessel	4 (7.8)
Myocardial infarction	1 (2.0)
Heart failure	2 (3.9)
Adverse event—road accident	1 (2.0)

Values are n (%). PCI, percutaneous coronary intervention; RPDA, right posterior descending descending artery; RPL, right posterolateral artery.

Twelve patients (23.5%) had complications after Solitaire use. Ten patients had distal embolization (19.6%), 1 patient had no reflow phenomenon, and 1 patient had embolization of thrombus from the right posterolateral artery to the right posterior descending artery. These were largely resolved with aspiration using aspiration catheter or with intracoronary medications.

There was no report of in-hospital complications. Within 30 days postdischarge, 7 patients (13.7%) had cardiac-related rehospitalizations; of these, 4 patients (7.8%) had planned admissions for revascularization of nonculprit vessels with 3 undergoing stage PCI and 1 patient undergoing coronary artery bypass grafting. Two patients (3.9%) were admitted for heart failure, and 1 patient (2%) was readmitted for atypical chest pain with mild troponin elevation and no electrocardiographic changes. Repeat coronary angiogram with optical coherence tomography was performed, which demonstrated patent stent in the IRA with no residual thrombus, new plaque rupture or evidence of vessel injury. A diagnosis of possible vasospasm was given. One patient was hospitalized following a road traffic accident.

## Discussion

The Solitaire device, with a successful recanalization rate of 92.2% at the end of the procedure, was demonstrated to be effective and safe in the management of patients with ACS and recalcitrant large thrombus. In a cohort of 51 patients with residual large thrombus despite treatment using conventional means, use of the Solitaire device as an adjunctive tool resulted in improvement in TIMI flow with 39.2% of patients having TIMI 0 to 1 flow at the start of the procedure to 100% of patients achieving TIMI 2 to 3 flow at the end of the procedure. There was a significant reduction in thrombus burden from TG 4 in 51 patients at the start of procedure to TG of ≤2 in 28 (54.9%) of patients immediately after Solitaire and 43 (84.3%) patients by the end of the procedure. MBG also improved at a rate of 40% after Solitaire use and 66.7% at the end of the procedure.

The use of the Solitaire device in the coronary arteries was found to be safe with no occurrence of stroke in our cohort. The most common intraprocedural adverse event was from distal embolization after Solitaire use, affecting 10 (19.6%) patients. This resolved after thrombus aspiration and use of intracoronary vasodilators. There was no incidence of vessel spasm, dissection, or perforation in our cohort. The device was successfully deployed and retrieved in all patients.

The use of stent retrievers is used routinely for the retrieval of thrombus in patients with acute ischemic stroke to restore cerebral perfusion. Case reports and case series have described the use of the Solitaire and Trevo (Stryker Neurovascular) devices to retrieve large coronary artery thrombus.^10^^,^^14^ Spirito et al^11^ demonstrated safety and efficacy of the NeVa thrombectomy device as primary therapy for large thrombus in patients with ACS.^11^ This is the first multicenter study to demonstrate the efficacy and safety of the Solitaire device as a bailout strategy in a patient population that proved difficult to treat by conventional methods. In the article by Spirito et al,^11^ a guide extension catheter was used in conjunction with the stent retriever to facilitate aspiration. In this study, measures taken to minimize the risk of stroke included ensuring the guide catheter was well seated and engaging the coronary ostium, and applying continuous aspiration on the guide catheter and microcatheter during retrieval of the Solitaire, appeared to be effective. Distal embolization occurred in 19.6% of patients, but there was angiographic improvement in most patients with the administration of intracoronary vasodilators and thrombus aspiration. While distal embolization is common when intervening on a lesion with high thrombus burden, a close tip stent design or filter could potentially reduce the incidence of distal embolization of large thrombus as demonstrated in an in vitro model.^15^

A large number of patients (80.4%) recruited in the study returned for use of the Solitaire device after deferring PCI. This was largely to allow time for consent to be taken before the use of the Solitaire device. It would have been more ideal to assess the efficacy of the device during the primary procedure and would have conferred a bigger benefit to the patient possibly negating the need for a second procedure. Because patients with stenosis and calcific lesions proximal to the thrombus and grafted vessels were excluded from this study, the safety and efficacy of the Solitaire device in vessels with complex anatomy and bypass grafts is not known.

This first-in-human study on the use of the Solitaire device in the coronary vessels demonstrates that it can be an effective adjunctive tool in the management of refractory coronary thrombus not amendable to treatment by conventional methods. Together with a low risk of clinically relevant procedural complications, this study supports its use alongside existing devices for thrombus management in the coronary vasculature. This study adds to the current literature on the potential use of stent retrieval devices in the coronary arteries in the treatment of large intracoronary thrombus. Future studies assessing its superiority over conventional methods in the management of large coronary thrombus as well as clinical outcome data are warranted.

### Limitations

This was a single-arm study with a limited patient cohort. Evidence obtained is therefore preliminary, and randomized control data are needed to confirm the efficacy of the device compared with that of standard treatment. The device demonstrated reduced effectiveness in very large ectatic vessels, as indicated by the findings where 5 patients (mean vessel size of 7.0 mm) still had a residual thrombus burden of grade 4 at the end of procedure. This suggest that the current generation of the Solitaire device may not be optimal for larger vessel sizes (Supplemental Figure S1). As patients with calcified vessels with significant proximal stenosis and bypass grafts were excluded in the study, the safety and efficacy of the Solitaire device in such patients is not known. A large number of patients had use of the Solitaire device at deferred stage. It would be beneficial to assess the efficacy of the device during the primary procedure.

## Conclusions

This single-arm multicenter prospective study has demonstrated the efficacy of the Solitaire device as an adjunctive tool in the treatment of recalcitrant intracoronary thrombus, with a low risk of clinically relevant complications. These data support further investigation of this device in randomized controlled trials against current standards of care to establish the role of stent retrieval technology in the treatment of large thrombus in the coronary vasculature.

## References

[1] Fokkema M.L., Vlaar P.J., Svilaas T. (2009). Incidence and clinical consequences of distal embolization on the coronary angiogram after percutaneous coronary intervention for ST-elevation myocardial infarction. Eur Heart J.

[2] Svilaas T., Vlaar P.J., van der Horst I.C. (2008). Thrombus aspiration during primary percutaneous coronary intervention. N Engl J Med.

[3] Burzotta F., Trani C., Romagnoli E. (2005). Manual thrombus-aspiration improves myocardial reperfusion: the randomized evaluation of the effect of mechanical reduction of distal embolization by thrombus-aspiration in primary and rescue angioplasty (REMEDIA) trial. J Am Coll Cardiol.

[4] Silva-Orrego P., Colombo P., Bigi R. (2006). Thrombus aspiration before primary angioplasty improves myocardial reperfusion in acute myocardial infarction: the DEAR-MI (Dethrombosis to Enhance Acute Reperfusion in Myocardial Infarction) study. J Am Coll Cardiol.

[5] Jolly S.S., Cairns J.A., Yusuf S. (2015). Randomized trial of primary PCI with or without routine manual thrombectomy. N Engl J Med.

[6] Fröbert O., Lagerqvist B., Olivecrona G.K. (2013). Thrombus aspiration during ST-segment elevation myocardial infarction. N Engl J Med.

[7] Fanous A.A., Siddiqui A.H. (2016). Mechanical thrombectomy: stent retrievers vs. aspiration catheters. Cor Vasa.

[8] Saver J.L., Jahan R., Levy E.I. (2012). Solitaire flow restoration device versus the Merci Retriever in patients with acute ischaemic stroke (SWIFT): a randomised, parallel-group, non-inferiority trial. Lancet.

[9] Uribe C.E., Zuniga M., Madrid C. (2016). Mechanical thrombectomy using the Solitaire stent in a left main coronary artery: a novel approach to coronary thrombus retrieval. Catheter Cardiovasc Interv.

[10] Khoo D.Z.L., Lee J.H., Watson T.J., Ong P.J.L. (2019). The Solitaire device—on the cards for retrieval of recalcitrant thrombus in acute coronary syndrome. EuroIntervention.

[11] Spirito A., Quagliana A., Coiro M. (2022). A prospective, first-in-human use of the NeVa mechanical thrombectomy device for patients with acute coronary syndromes. EuroIntervention.

[12] Garcia-Garcia H.M., McFadden E.P., Farb A. (2018). Standardized end point definitions for coronary intervention trials: the Academic Research Consortium-2 Consensus Document. Eur Heart J.

[13] Simon R. (1989). Optimal two-stage designs for phase II clinical trials. Control Clin trials.

[14] Bhoopalan K., Rajendran R., Alafarsamy S., Keavamoorthy N. (2019). Successful extraction of refractory thrombus from an ectatic coronary artery using stent retriever during primary angioplasty for acute myocardial infarction: a case report. Eur Heart J Case Rep.

[15] Li J., Tiberi R., Bhogal P. (2024). Impact of stent-retriever tip design on distal embolization during mechanical thrombectomy: a randomized in vitro evaluation. J Neurointerv Surg.

