# Supplementary material for: Efficacy and Safety of Solitaire Revascularization Device in Managing Refractory Thrombus in Acute Coronary Syndrome
Source: J Soc Cardiovasc Angiogr Interv. 2025 Nov 11;4(12):104004. doi: 10.1016/j.jscai.2025.104004 (PMC12766044; doi:10.1016/j.jscai.2025.104004)
Supplement: Supplementary Material [file mmc1.docx]

**Supplementary data**

Supplementary Table 1. Solitaire Platinum stent sizes.


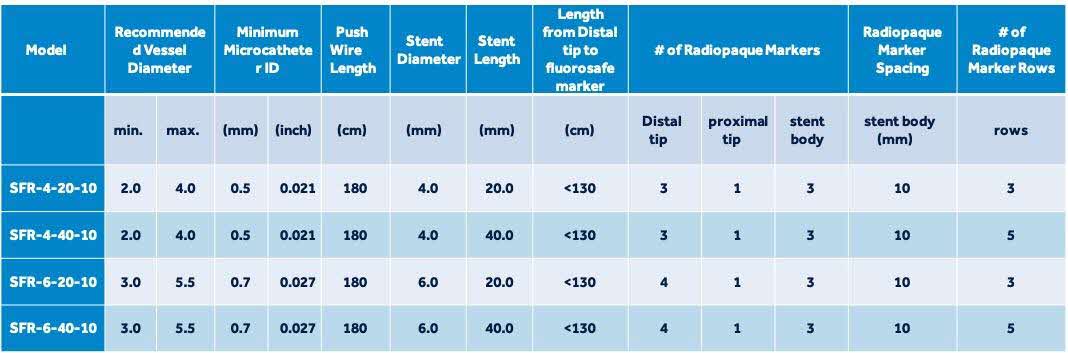


Supplementary Table 2. Angiographic outcomes

| Score/Grade | Description |
| --- | --- |
| TIMI Flow | |
| 0 | No perfusion: No antegrade flow beyond the point of occlusion. |
| 1 | Penetration without perfusion: The contrast material passes beyond the area of obstruction, but “hangs up” and fails to opacify the entire coronary bed distal to the obstruction for the duration of the cine run. |
| 2 | Partial reperfusion: The contrast material passes across the obstruction and opacifies the coronary bed distal to the obstruction However, the rate of entry of contrast into the vessel distal to the obstruction and/or its rate of clearance from the distal bed are perceptibly slower than its entry into and/or clearance from comparable areas not perfused by the culprit vessel |
| 3 | Complete perfusion: Antegrade flow into the bed distal to the obstruction occurs as promptly as into the bed proximal to the obstruction and clearance of contrast material from the involved bed is as rapid as from an uninvolved bed in the same vessel or the opposite artery. |
| TIMI THROMBUS GRADE | |
| 0 | No cineangiographic characteristics of thrombus are present |
| 1 | Possible thrombus is present, with such angiography characteristics as reduced contrast density, haziness, irregular lesion contour, or a smooth convex (meniscus) at the site of total occlusion suggestive but not diagnostic of thrombus |
| 2 | Definite thrombus, with greatest dimensions <0.5 the vessel diameter |
| 3 | Definite thrombus but with greatest linear dimension >0.5 but <2 vessel diameters |
| 4 | Definite thrombus, with the largest dimension >2 vessel diameters |
| 5 | Total occlusion |
| Myocardial blush grade | |
| 0 | No myocardial blush or contrast density |
| 1 | Minimal myocardial blush or contrast density |
| 2 | Moderate myocardial blush or contrast density but less than that obtained during angiography of a contralateral or ipsilateral non–infarct-related coronary artery |
| 3 | Normal myocardial blush or contrast density, comparable with that obtained during angiography of a contralateral or ipsilateral non–infarct related coronary artery |


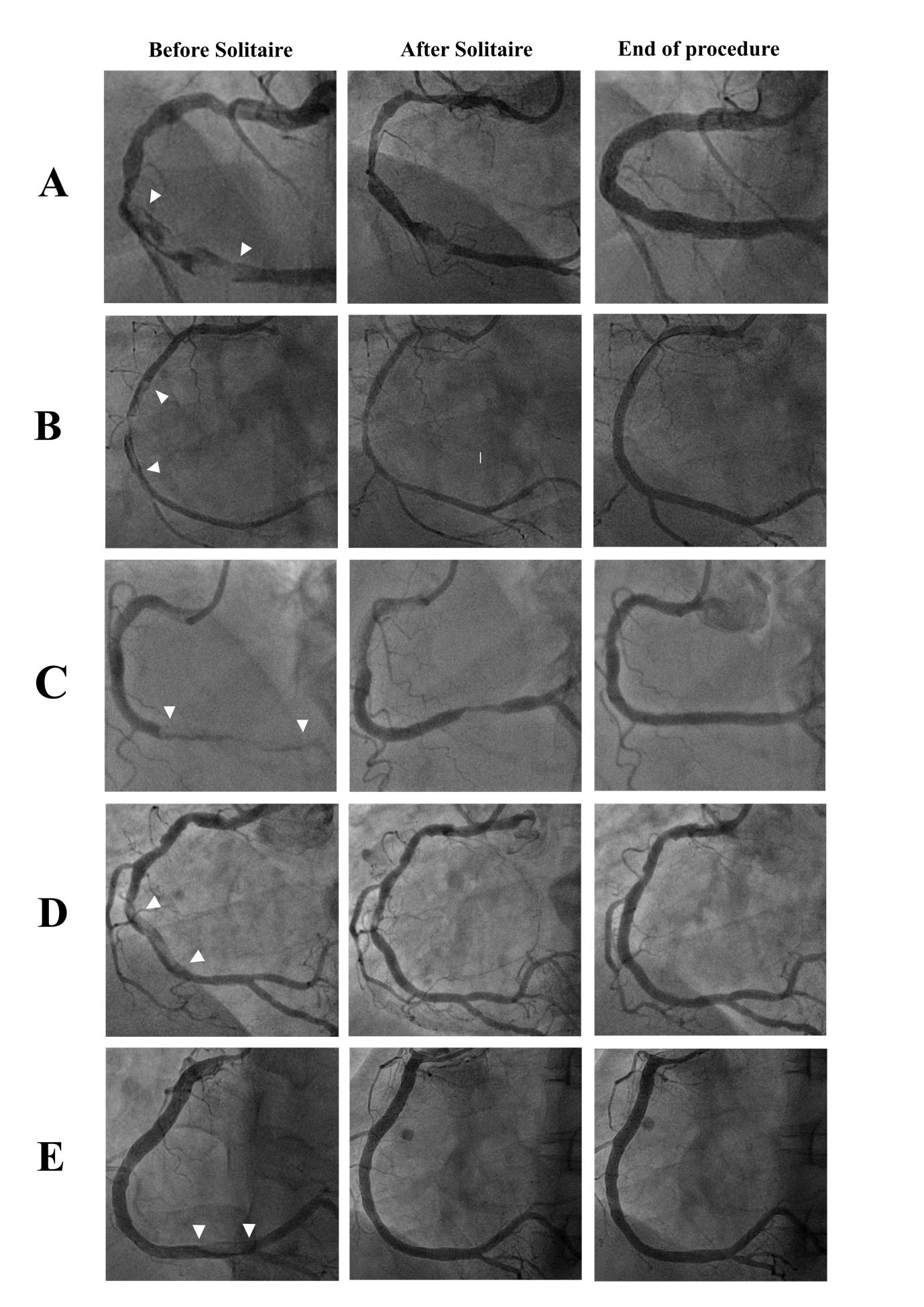


Supplementary Figure 1. Angiographic outcomes showing large thrombus before Solitaire deployment, reduction in thrombus burden after Solitaire deployment and final result at end of procedure.
